# Novel findings on the impact of chytridiomycosis on the cardiac function of anurans: sensitive vs. tolerant species

**DOI:** 10.7717/peerj.5891

**Published:** 2018-11-07

**Authors:** Raquel F. Salla, Gisele M. Rizzi-Possignolo, Cristiane R. Oliveira, Carolina Lambertini, Lilian Franco-Belussi, Domingos S. Leite, Elaine Cristina M. Silva-Zacarin, Fábio C. Abdalla, Thomas S. Jenkinson, Luís Felipe Toledo, Monica Jones-Costa

**Affiliations:** 1 Department of Biology, Universidade Federal de São Carlos, Sorocaba, SP, Brazil; 2 Department of Animal Biology, Universidade Estadual de Campinas, Campinas, SP, Brazil; 3 Department of Biology, University of California, Santa Cruz, Santa Cruz, CA, United States of America; 4 Department of Genetic, Evolution, Microbiology and Immunology, Universidade Estadual de Campinas, Campinas, SP, Brazil; 5 Department of Environmental Science, Policy, and Management, University of California, Berkeley, United States of America

**Keywords:** Chytrid, Frogs, Amphibian decline, Heart, Wildlife disease

## Abstract

**Background:**

Understanding of the physiological effects of chytridiomycosis is crucial to worldwide amphibian conservation. Therefore, we analyzed the cardiac function of two anuran species (*Xenopus laevis* and* Physalaemus albonotatus*) with different susceptibilities to infection by the causative agent of chytridiomycosis,* Batrachochytrium dendrobatidis* (hereafter *Bd*).

**Methods:**

We analyzed the *in situ* heart rate (*f*_*H*_ - bpm), relative ventricular mass (RVM -%), and Ca^2+^ handling in heart of *Bd* infected animals compared to uninfected controls of both study species.

**Results:**

*Bd* infection resulted in a 78% decrease in contraction force values in *P. albonotatus* when compared to the less susceptible *X. laevis*. This negative effect was even more evident (82%) for the cardiac pumping capacity. The time to reach peak tension was 125% longer in *P. albonotatus* than in *X. laevis*, and cardiac relaxation was 57% longer.

**Discussion:**

These results indicate a delay in the cardiac cycle of *P. albonotatus* on a beat-to-beat basis, which was corroborated by the bradycardia observed *in situ*. In summary, *Bd*-sensitive species present impaired cardiac function, which could be a factor in mortality risk. The more pronounced effects of *Bd* in *P. albonotatus* may not only result from electrolyte imbalance, as previously reported, but also could be an effect of toxins produced by *Bd*. For *X. laevis*, the ability to promote cardiac adjustments seems to be an important homeostatic feature that allows greater tolerance to chytridiomycosis. This study provides new physiological mechanisms underlying the tolerance or susceptibility of amphibian species to chytridiomycosis, which determine their adaptability to survive in the affected environments.

## Introduction

Belonging to an early diverging clade of Fungi (Schaechter et al., 2013), the pathogen *Batrachochytrium dendrobatidis* (*Bd*) has been identified as one of the main causes of amphibian declines and extinctions worldwide (Schloegel et al., 2006; Kriger & Hero, 2007; Fisher, Garner & Walker, 2009; Farrer et al., 2011; Lips, 2016). Understanding the physiological interactions between amphibian hosts and this pathogen is crucial to conserve global biodiversity (James et al., 2015). Although there have been previous investigations into chytridiomycosis pathophysiology (e.g., Voyles, Rosenblum & Berger, 2011; Voyles et al., 2012; Salla et al., 2015; Bovo et al., 2016), the mechanistic process underlying this pathogenesis remain unresolved.

Physiologically, chytridiomycosis is known to lead to hyperkeratosis which impairs skin function in adult frogs (Kriger & Hero, 2007; Voyles et al., 2009; Carver, Bell & Waldman, 2010; Bovo et al., 2016; Brannelly et al., 2017). Considering that amphibian skin is substantially implicated in the regulation of osmotic balance (electrolyte and water balance) (Ferreira & Hill, 1978; Wright & Whitaker, 2001), this pathophysiological damage is particularly problematic for hosts since many metabolic processes are tightly dependent on skin-mediated homeostatic mechanisms (Shoemaker et al., 1992; Boutilier et al., 1997).

Plasma osmolality and electrolyte concentration are disturbed in *Bd*-infected adult frogs (e.g., hyponatremia and hypokalemia) (Voyles et al., 2007; Voyles et al., 2009; Marcum et al., 2010), causing asystole and cardiac arrest (Voyles et al., 2009; Voyles et al., 2012). Moreover, as initially suggested by Blaustein et al. (2005) and recently confirmed (Brutyn et al., 2012; Fites et al., 2013), *Bd* can also produce toxins that could affect multiple organs. These prior studies provide clear evidence that *Bd* infection can affect the cardiac function of anurans. However, electrolyte-dependent and toxin-based mechanisms are not mutually exclusive for this disease. To date, there is not sufficient evidence to determine which is the critical process by which *Bd* infection alters the cardiac performance of amphibians in a species-specific manner (Salla et al., 2015).

The exact reason why, and to what extent, these species present different levels of tolerance to *Bd* remains uncertain, but interspecific variability in physiological responses to *Bd* may explain the differences in mortality rates among *Bd*-infected amphibian species. Our previous study (Salla et al., 2015) demonstrated that *Bd* infected bullfrog tadpoles present an elevation of the cardiac contraction force correlated to a myocardial hypertrophy. Such effects on cardiac performance demonstrate that bullfrog tadpoles develop physiological adaptations in order to avoid the lethal effects of the fungus. This represents a possible interspecific feature of tolerance. In this current investigation, we aimed to determine whether *X. laevis*, a *Bd*-tolerant species with cutaneous innate immune defenses (Woodhams et al., 2006; Rollins-Smith et al., 2009), also depends on cardiac adjustments to survive *Bd* infection. Studies on *Bd* tolerant species are important in order to understand *Bd* dynamics, as tolerant species may exhibit compensatory mechanisms that are not seen in sensitive species.

Considering the previous reports and the general paucity of studies analyzing the effects of *Bd* on amphibians’ physiology, this study aimed to evaluate and compare the outcomes of *Bd*-infection in two anuran species with different sensitivities to chytridiomycosis: the African clawed frog, *Xenopus laevis*, and the meowing frog, *Physalaemus albonotatus*. Deepening our knowledge on chytridiomycosis infection is fundamental for the consolidation of effective action against the ecological impacts of this fungus. In addition, we also provide information about possible adaptive strategies that can determine the survival of amphibians in their natural habitats.

## Materials & Methods

### Collection and animal care

Male individuals of *X. laevis* (*n* = 18; average body mass = 4.51 ± 0.24 g) were obtained from a commercial establishment (Divine Poultry, Sorocaba, São Paulo, Brazil). Male Individuals of *P. albonotatus* (*n* = 13, average body mass = 2.60 ± 0.07 g) were collected in the wild (Brazilian Institute of Environment—IBAMA collecting permit #18566-1; Sisgen register number - A9A742E), in the surroundings of the hotel Caminhos do Pantanal Inn, district of Albuquerque, Mato Grosso do Sul, Brazil. This region was selected because there are no records of *Bd* thus far (James et al., 2015), which avoids the use of previously infected specimens in the experiments. Once transported to the Laboratory of Conservation Physiology of the Federal University of São Carlos (State of São Paulo, city of Sorocaba, Brazil), the animals were maintained in the laboratory for 20 days to recover from stress related to transportation and handling, and to promote acclimatization. Individuals of *X. laevis* (aquatic species) were kept in 10 L aquaria (1 animal/L) and fed daily with fish feed containing 30% crude protein (Alcon R) (Haislip et al., 2011). Individuals of *P. albonotatus* (semi-aquatic species) were kept in 10 L terraria (1 animal/dm^3^) containing 1 L of dechlorinated water to maintain humidity, and fed daily with larvae of the citrus fruit borer (*Ecdytolopha aurantiana*, Tortricidae, Lepidoptera) (Uetanabaro et al., 2008).

The water of the aquaria was dechlorinated, constantly aerated, and the animals were maintained at a controlled temperature (20 °C) and natural photoperiod (∼12 h light: 12 h dark). Water was monitored daily to ensure that the physical and chemical parameters were kept at acceptable levels (pH 7.1–7.3; hardness as CaCO_3_ 48–58 mg L^−1^; dissolved oxygen 6.8–7.4 mg L^−1^; conductivity 99.77 µS cm^−1^), similar to most Brazilian inland waters (Conselho Nacional do Meio Ambiente-CONAMA, 2011). Ammonia concentration in water was monitored every day (K-1510;CHEMets), and remained below 1.1 mg L^−1^. All physical and chemical parameters fufil the conditions of the ASTM (2004) guidelines.

Soon after the arrival of the animals in the laboratory, diagnosis to detect previous *Bd* infection was carried out in both species. All individuals were swabbed (Kriger et al., 2006; Hyatt et al., 2007; Lambertini et al., 2013), and the samples were tested for *Bd* using Taqman quantitative PCR (qPCR) (Boyle et al., 2004; Lambertini et al., 2013). Analyzes confirmed that no animal had prior *Bd* infection.

### Treatments

Individuals of *X. laevis* (*n* = 18) were divided into two aquariums (treatments): the infected aquarium (*Bd*^+^; *n* = 9) and the control aquarium (*Bd*^−^; *n* = 9). Both *Xenopus* aquariums (35 × 21 × 25 cm) were filled with 5L of dechlorinated water each. Individuals of *P. albonotatus* (*n* = 13) were also divided into two aquariums (35 × 21 × 25 cm): the infected aquarium (*Bd*^+^; *n* = 7) and the control aquarium (*Bd*^−^; *n* = 6). Both *Physalaemus* aquariums were filled with 1.0 L of dechlorinated water. Such amount was sufficient to keep the ventrum of the animals in contact with water, without submerging them completely. Throughout the entirety of the experiments (from acclimatization to the end of infection), the Physalaemus aquariums remained slightly inclined in order to allow the animals to transit between water and a humid area without restrictions.

For the infection of *Bd*^+^ groups we used strain CLFT 023 strain provided by LaNHAB (Institute of Genetics, Evolution and Bioagents, UNICAMP Institute of Biology, Campinas), descendant from a pure culture grown in broth 1% tryptone for 7 days (at 11 °C), and propagated in Petri dishes containing 1% tryptone agar (Longcore, Pessier & Nichols, 1999). This strain belongs to the global panzootic lineage (*Bd*-GPL), a highly virulent lineage, implicated in cases of amphibian declines worldwide (Farrer et al., 2011; Rosenblum et al., 2013). We grew the culture (at 23 °C) for 7 days, then collected zoospores using two ml of distilled water. An aliquot of the spore suspension was counted in a Neubauer chamber to estimate the concentration of zoospores. After that, 50 mL of *Bd* inoculum (1.3 ×  10^7^ zoospores/mL) was added to the *Physalaemus Bd*^+^ aquarium (previously containing 950 mL of dechlorinated water), and 250 mL of the same *Bd* inoculum to the *Xenopus Bd*^+^ aquarium (previously containing 4,750 mL of dechlorinated water), to reach a final concentration of 6.5 ×10^5^ zoospores /mL in each inoculated aquarium. This concentration is consistent with the inoculum load used by other researches investigating chytrid infection in laboratory conditions (Savage & Zamudio, 2011; Gervasi et al., 2013; Salla et al., 2015; Venesky et al., 2015; Bovo et al., 2016). Both species remained under the same conditions for one week, monitored and fed daily, with no water change. After the seventh day of infection, confirmatory diagnosis was carried out through qPCR as described above (Boyle et al., 2004; Lambertini et al., 2013) with CLFT 023 standards of 0.1, 1, 10, 100, and 1,000 zoospore genomic equivalents (GE) to determine the infection intensity of *Bd* in each amphibian host. The zoospore standards were prepared with using the same inoculation strain, CLFT 023.

The present experimental design was based on prior ecophysiological studies on chytrid infection with cohoused experimental groups (Bovo et al., 2016;  Blaustein et al., 2018; Gabor et al., 2018; Robak, Corinne & Richards-Zawacki, 2018). The maintenance of animals from each experimental group (infected / not infected) in a single aquarium may initially be construed as a dependency of samples. However, we treated the different levels of infection (*Bd* loads) as independent variables for the analyses. Therefore, our analysis of the physiological responses from uninfected (*Bd*^−^) to infected animals (*Bd*^+^) according to their individual *Bd* loads circumvents the problem of sample interdependency.

At the end of the infection period, animals were euthanized by sectioning the spinal cord and decerebration. This procedure follows the ethics statements proposed by the (AVMA, 2007) and the (CONCEA, 2013). The animal experimentation ethics committee of the Federal University of São Carlos approved all the procedures of this experiment (permit CEUA #4225040612) in accordance with Brazilian laws. After euthanasia, the abdominal region was surgically opened in a caudal-cranial direction for pericardium exposure. The cardiac rate *(in situ f*_H_) of both experimental groups (*Bd*^−^ and *Bd*^+^) from both species was determined visually (for 3 min) and expressed as beats per minute (bpm). Although this procedure does not exclude all the effects of adrenergic tonus, it avoids the side effects of anesthetics used for the insertion of ECG electrodes (Wassersug, Paul & Feder, 1981; Williams et al., 2017).

### Relative ventricular mass (RVM)

After measuring the *in situ f*_*H*_, the heart was dissected and the ventricle carefully separated. Ventricles from each experimental group (*Physalaemus Bd*^+^*: n* = 7*; Physalaemus Bd*^−^*: n* = 6*; Xenopus Bd*^+^*: n* = 9*; Xenopus Bd*^−^: *n* = 6) were weighed (Wv = ventricle weight) and the ventricular mass was expressed as a percentage of body mass (Wb = body weight; RVM - % of Wb).

### Analysis of the “in vitro” heart function

Ventricle strips (∼1.2 mm diameter; mass = 0.65 ± 0.1 mg; length = 1.2 ± 0.1 mm) from each experimental group (*Physalaemus Bd*^+^*: n* = 10*; Physalaemus Bd*^−^*: n* = 10*; Xenopus Bd*^+^*: n* = 9*; Xenopus Bd*^−^: *n* = 8) were prepared for the isometric contraction recordings following the procedures developed by Costa et al. (2008). Each ventricle strip was transferred to a 20 mL thermostatic cuvet (25 ± 1 °C), and suspended with surgical thread. The thread was attached to an isometric force transducer (Letica Corporation, Oklahoma, OK, USA). The other end was tied around a platinum electrode. This electrode was connected to a stimulator (AVS100D; Brazil). The contraction records were recorded using an acquisition software (AQCAD100; Brazil). During the stabilization period, the contraction force (CF: mN mm^−2^) was measured at the sub-physiological frequency of 0.2 Hz for 40 min. Thereafter, the stimulation frequency was increased stepwise in increments of 0.2 Hz until the frequency at which the muscle failed to show regular contractions was reached (maximal frequency). The maximal *in vitro* stimulation frequency was considered to be where at least 80% of the strips were still able to contract regularly (i.e., the stimulation rate at which every stimulation elicited contraction). During the force-frequency protocols, we analyzed the cardiac force (CF: mN mm^−2^), the cardiac pumping capacity (CPC: mN mm^−2^ min^−1^); and the chronotropic parameters; time to peak tension (TPT –ms) and the time to half relaxation (THR –ms). The cardiac pumping capacity is the product of the stimulation frequency and the twitch force (Matikainen & Vornanen, 1992). All these parameters were measured with the following sample sizes: *Physalaemus Bd*^+^∕*n* = 10; *Physalaemus* Bd^−^*/n* = 10*; Xenopus Bd*^+^*/n* = 9*; Xenopus Bd*^−^/*n* = 8.

### Statistical analysis

The results are represented as means ± S.E (standard error). The data were first analyzed with the Bartlet test to confirm the homogeneity of variances. The Shapiro–Wilk and Kolmogorov–Smirnov tests were used to evaluate the normality of the samples. Then, to compare the results obtained among the stimulation frequencies of the contractility parameters (e.g., 0.2 to 1.6 Hz–FC, TPT and THR) we ran the parametric Tukey-Kramer multiple comparisons test. Dunnett’s test was employed for both comparisons between Bd^−^ and Bd^+^ groups of the same species, and for comparisons between the two species. Finally, to compare the heart frequency and the relative ventricular mass between Bd^−^ and Bd^+^ groups of the same species we ran the Unpaired *T*-test (Software GraphPad Instat version 3.00; GraphPad Software, La Jolla, CA, USA). Differences between results at 5% level (*P* < 0.05) were considered significant. To correlate the infection load and its effect on the contraction force of each individual, we plotted the respective points on a graph and applied the appropriate trend line (including the equation of the line).

## Results

After the fifth day of infection, the first symptoms we observed in *Bd*^+^ groups were the rejection of food in both species, and lethargy only in *P. albonotatus*. Regarding the *in situ* analyses for *P. albonotatus*, a bradycardia was observed (*P* = 0.041) in the *Bd*^+^ group (116 ± 2 bpm) when compared to control animals (*Bd*^−^ = 136 ± 3) (Table 1). The relative ventricular mass of *P. albonotatus* (RVM: % body mass) was lower (*P* = 0.03), with atrophy of the heart muscle in individuals infected with *Bd* (*Bd*^+^: 0.188 ± 0.004) when compared to the controls (*Bd*^−^:0.263 ± 0.005) (Table 1).

**Table 1:** Relative ventricular mass and heart rate. Mean values of relative ventricular mass (RVM: % of body mass) and heart rate (*f*_H_ - bpm) of *P. albonotatus* and *X. laevis* in response to *Bd* infection (*Bd*^+^) and their respective controls (*Bd* ^−^). Mean ± S.E (standard error).

		*f*_**H**_ (bpm)		RVM (%)	
Species	Group	Mean	S.E.	Mean	S.E.
*Physalaemus albonotatus*	*Bd*^−^	136		3	0.28		0.01
*Physalaemus albonotatus*	*Bd*^+^	116^*^	2	0.15^*^	0.01
*P*-value		*P* < 0.0001		*P* < 0.0001	
*Xenopus laevis*	*Bd*^−^	84		6	0.10		0.01
*Xenopus laevis*	*Bd*^+^	84	6	0.19^*^	0.02
*P*-value		*P* > 0.05		*P* < 0.0001	

**Notes.**

* Indicates significant differences between groups (Unpaired T-test -*P* < 0.05/*P* < 0.0001), while “NS” denotes not significant difference (*P* > 0.05).

For *X. laevis*, however, there were no significant changes in heart rate (*f*_H_, Table 1) between the infected animals (*Bd*^+^: 84 ± 6 bpm) and the control group (*Bd*^−^: 84 ± 6 bpm). On the other hand, the relative ventricular mass of infected *X. laevis* was higher (*Bd*^+^: 0.19 ± 0.02%) when compared to those obtained for the control group (*Bd*^−^: 0.10 ± 0.01%) (Table 1).

**Figure 1:**
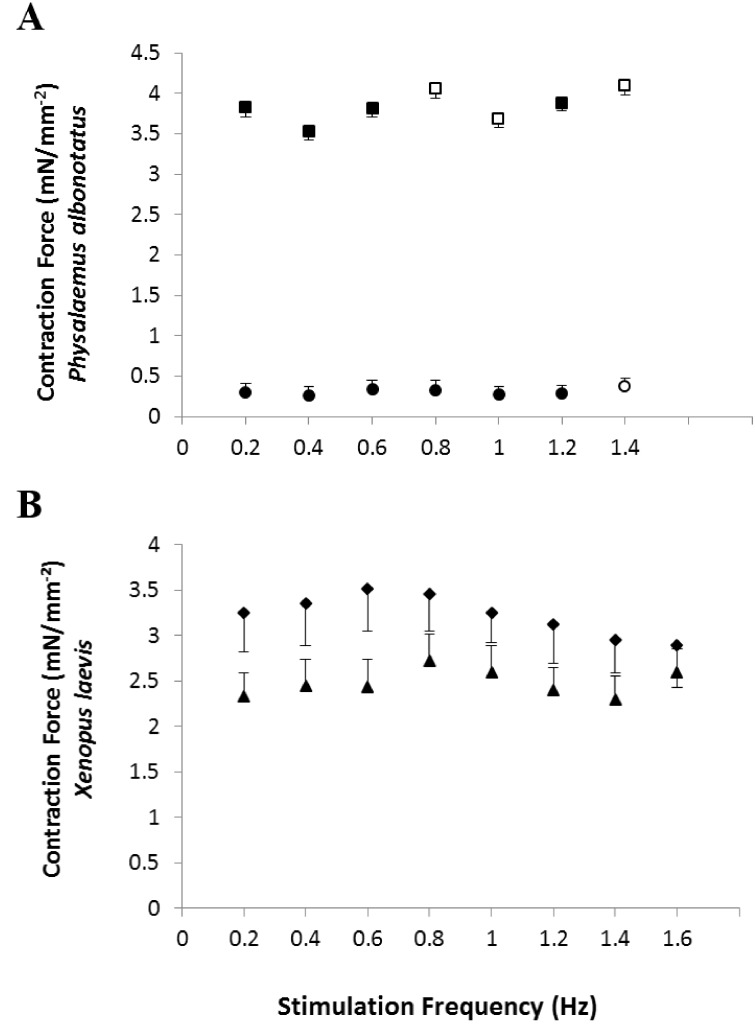
Effects of chytridiomycosis on the Contraction Force. Effects of increasing stimulation frequency on the contraction force (CF: mN mm^−2^) developed by the ventricular strips of *P. albonotatus* (1A; *Bd*^−^: *n* = 10, and *Bd*^+^: *n* = 10) and *X. laevis* (1B; *Bd*^−^ *n* = 8, and *Bd*^+^: *n* = 9). The point values represent mean ±  S.E. Blank markers denote significant difference (*P* < 0.05) between the CF developed by the same species in relation to the initial frequency (0.2 Hz). Significant differences (*P* < 0.05) between different treatments (*Bd*^−^ and *Bd*^+^) are represented by an asterisk “*” above the error bars of the *Bd*^+^ group.

*In vitro*, the contraction force (CF) developed by the ventricular strips of infected *P. albonotatus* (*Bd*^+^) was markedly reduced (∼40%; *P* = 0.028) when compared to their controls (*Bd*^−^) at all the frequencies (from 0.2 to 1.4 Hz - Fig. 1A). In contrast, for *X. laevis*, no differences were observed (*P* > 0.05) between the CF developed by ventricular strips of *Bd*^+^ and *Bd*^−^ groups at any stimulation frequency (Fig. 1B).

**Figure 2:**
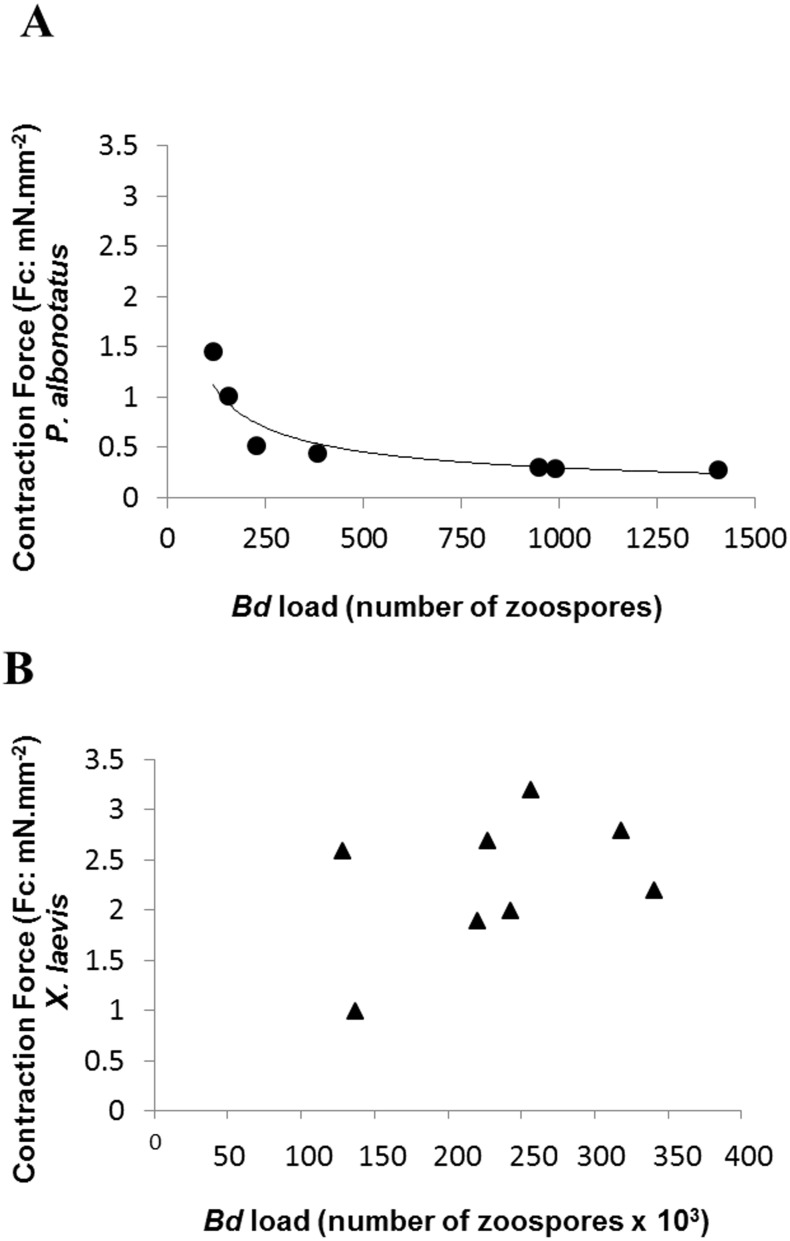
Correlation: *Bd* load vs. Contraction Force. Correlation between the *Bd* load acquired by each specimen and the contraction force (CF: mN mm^−2^) developed by the respective ventricular strips (at 0.2 Hz of stimulation frequency) of *P. albonotatus* (A: Line equation *y* = 20.986 ×  − 0.617; *r*^2^ = 0.8999) and *X. laevis* (note that there was no correlation for this species).

Although the infected groups of both species had been inoculated with the same concentration of zoospores, qPCR analysis showed that *Bd* load acquired by *P*. *albonotatus was* lower than the load observed for *X. laevis* (Table 2). Nevertheless, the effects of infection in *P. albonotatus* were more pronounced than in *X. laevis* (Fig. 2). There is a high correlation between the number of zoospores and the degree of reduction of the CF at 0.2 Hz in *P. albonotatus* (Fig. 2A); while for *X. laevis* this correlation was not significant (Fig. 2B).

**Table 2:** Diagnosis and quantification of *Bd* infection in *P. albonotatus* and *X. laevis* by means of qPCR analysis.

Species	Group	Individual/ catalogued number	Result	Zoospore number/load
*Physalaemus albonotatus*	*Bd*^+^	SLFT1134	Positive	116
*Physalaemus albonotatus*	*Bd*^+^	SLFT1135	Positive	946
*Physalaemus albonotatus*	*Bd*^+^	SLFT1136	Positive	1,403
*Physalaemus albonotatus*	*Bd*^+^	SLFT1137	Positive	989
*Physalaemus albonotatus*	*Bd*^+^	SLFT1138	Positive	301
*Physalaemus albonotatus*	*Bd*^+^	SLFT1139	Positive	224
*Physalaemus albonotatus*	*Bd*^−^	SLFT1140	Negative	X
*Physalaemus albonotatus*	*Bd*^−^	SLFT1141	Negative	X
*Physalaemus albonotatus*	*Bd*^−^	SLFT1142	Negative	X
*Physalaemus albonotatus*	*Bd*^−^	SLFT1143	Negative	X
*Physalaemus albonotatus*	*Bd*^−^	SLFT1144	Negative	X
*Physalaemus albonotatus*	*Bd*^−^	SLFT1145	Negative	X
*Xenopus laevis*	*Bd*^+^	SLFT1623	Positive	339928
*Xenopus laevis*	*Bd*^+^	SLFT1624	Positive	256466
*Xenopus laevis*	*Bd*^+^	SLFT1625	Positive	136492
*Xenopus laevis*	*Bd*^+^	SLFT1626	Positive	226856
*Xenopus laevis*	*Bd*^+^	SLFT1627	Positive	317924
*Xenopus laevis*	*Bd*^+^	SLFT1628	Positive	128004
*Xenopus laevis*	*Bd*^+^	SLFT1629	Positive	220239
*Xenopus laevis*	*Bd*^−^	SLFT1631	Negative	X
*Xenopus laevis*	*Bd*^−^	SLFT1615	Negative	X
*Xenopus laevis*	*Bd*^−^	SLFT1616	Negative	X
*Xenopus laevis*	*Bd*^−^	SLFT1617	Negative	X
*Xenopus laevis*	*Bd*^−^	SLFT1618	Negative	X
*Xenopus laevis*	*Bd*^−^	SLFT1619	Negative	X

**Figure 3:**
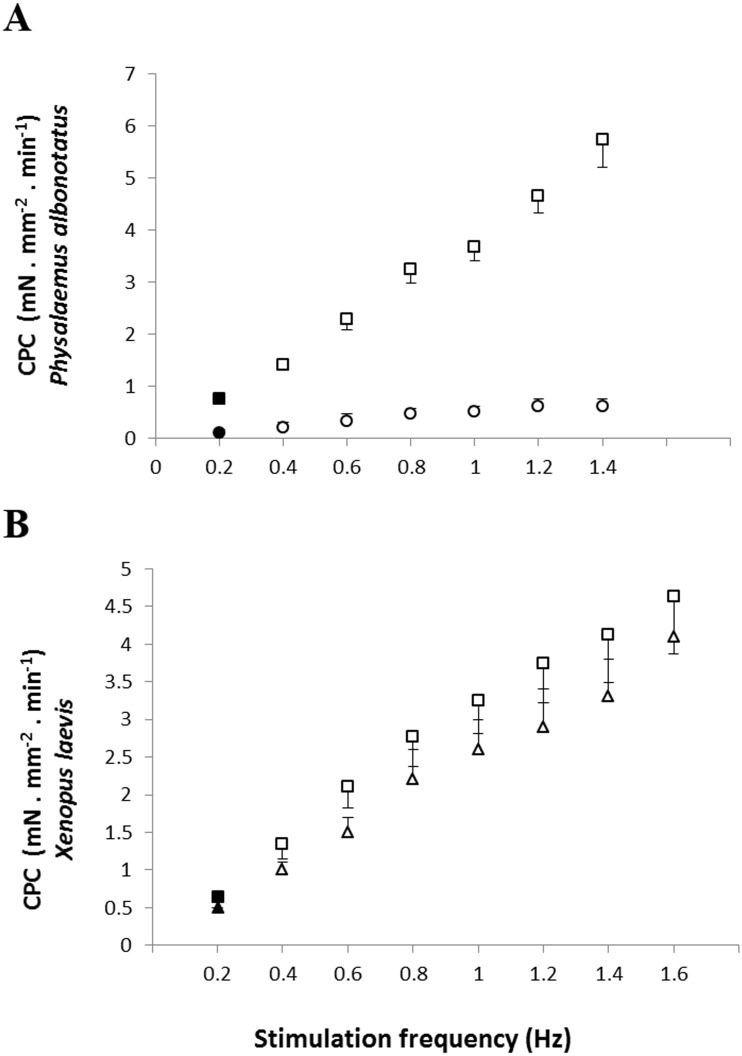
Cardiac pumping capacity of control and infected animals. Effect of the successive increase in stimulation frequencies on the cardiac pumping capacity (CPC: mN mm^−2^ min^−1^) developed by the ventricular strips of *P. albonotatus* (A: squares represent *Bd*^−^ group - *n* = 10, and circles represent *Bd*^+^ group - *n* = 10) and *X. laevis* (B: squares represent *Bd*^−^ group - *n* = 8, and triangles represent *Bd*^+^ group : *n* = 9). Points represent the means ± S.E. White markers denote significant difference (Tukey-Kramer test - *P* < 0.05) between the CPC developed by the same species at each stimulation frequency in relation to the initial frequency (0.2 Hz). Significant differences (Dunnett’s test - *P* < 0.05) between different treatments (*Bd*^−^ and *Bd*^+^) were observed only in *P. albonotatus*, in all stimulation frequencies.

Regarding the cardiac pumping capacity (CPC), despite having increased in both experimental groups (*Bd*^−^ and *Bd*^+^) as the frequency was raised, CPC values of *Bd*^+^ in *P. albonotatus* were much lower than those observed in their controls (*Bd*^−^) (Fig. 3A). For *X. laevis*, considering that the CF developed in both treatments (*Bd*^+^ and *Bd*^−^) were similar, the CPC values, despite having increased stepwise as stimulation frequency was increased, did not differ between experimental groups (Fig. 3B).

**Figure 4:**
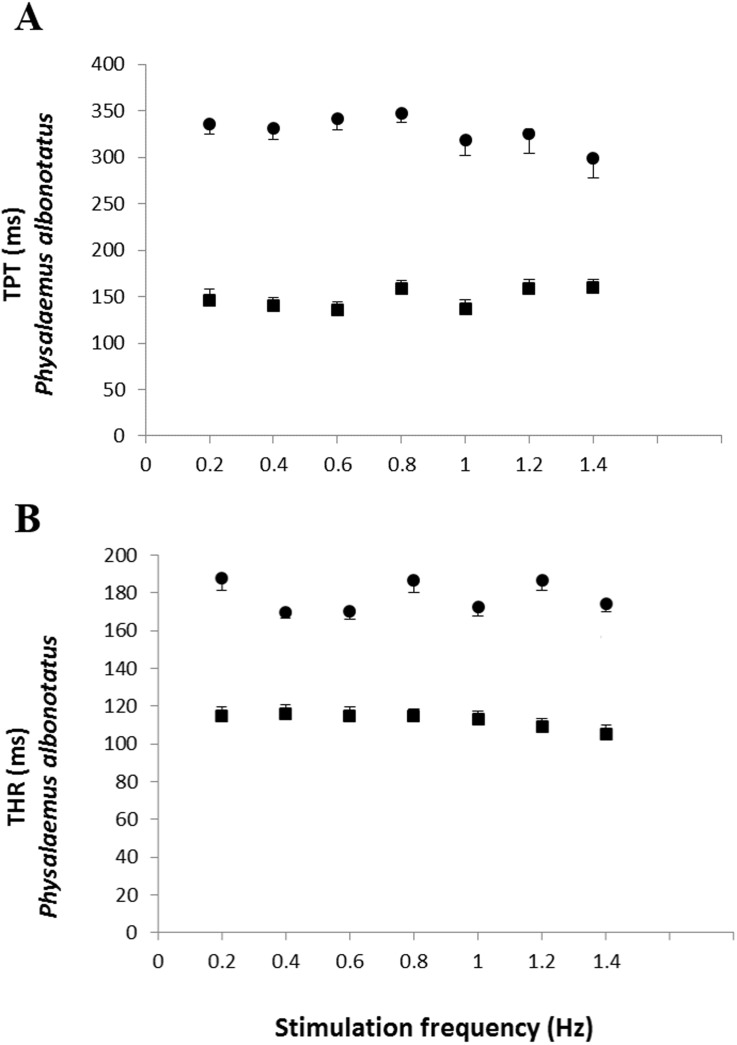
Time to peak tention and to relaxation of control and infected *P. albonotatus.* Effect of increased stimulation frequency over the time to reach peak tension (A: TPT - ms;) and the time to reach 50% of relaxation (B: THR - ms) of the ventricular strips of *P. albonotatus* (squares represent *Bd*^−^ group - *n* = 10, and circles represent *Bd*^+^* group - n* = 10**). The points represent mean values ±SE. Significant differences (Dunnett’s test - *P* < 0.05) between different treatments (*Bd*^−^ and *Bd*^+^) were observed in TPT and THR of *P. albonotatus*, in all stimulation frequencies. Note different scales in the figures.

**Figure 5:**
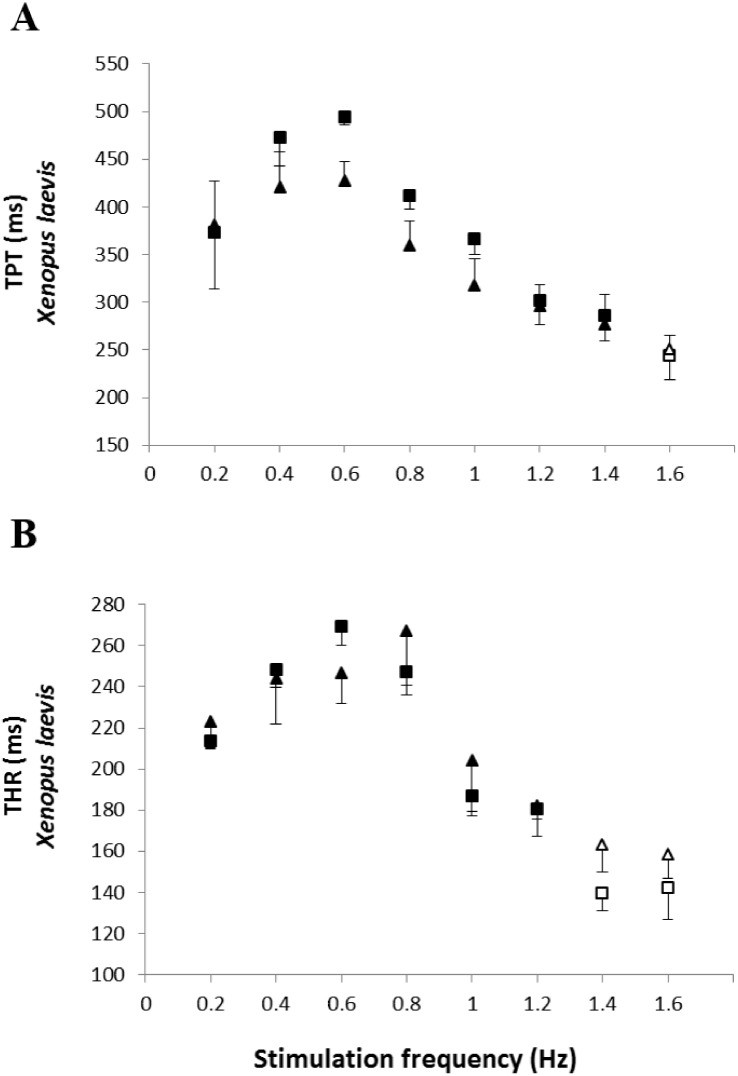
Time to peak tention and relaxation of heart strips os control and infected *X. laevis.* Effect of increased stimulation frequency over time to reach the peak tension (A: TPT - ms) and the time to reach 50% of relaxation (B: THR - ms) of the ventricular strips of *X. laevis* (squares represent *Bd*^−^ group - *n* = 8, and triangles represent *Bd*^+^ group - *n* = 9). The points represent the mean values ± S.E. White markers denote significant difference (Tukey-Kramer test - *P* < 0.05) between the TPT or THR developed by the same species at each stimulation frequency in relation to the initial frequency (0.2 Hz). There were no significant differences (Dunnett’s test - *P* > 0.05) between different treatments (*Bd*^−^ and *Bd*^+^) TPT and THR.

The analysis of the time-dependent parameters (TPT and THR) revealed that *Bd*^+^ *P. albonotatus* group had an elevation in both parameters (Fig. 4). For *X. laevis,* there were no differences between *Bd*^−^ and *Bd*^+^ groups at any time-dependent parameters, regardless of the stimulation frequency (Fig. 5).

**Figure 6:**
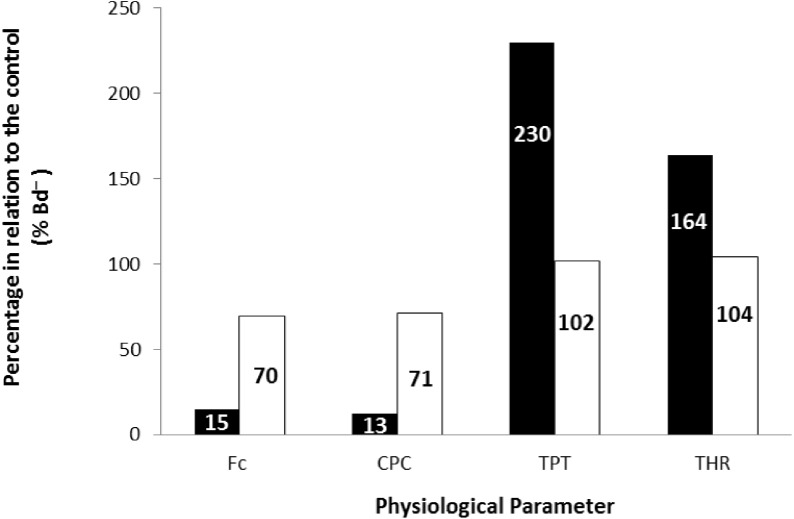
Comparative diagram of *Bd* infection effects in *P. albonotatus and X. laevis.* Comparative diagram of *Bd* infection (% of *Bd*^−^ at 0.2 Hz) of *P. albonotatus* (black bars) and *X. laevis* (white bars) in relation to the *in vitro* parameters (CF, CPC, TPT and THR). The values within the bars represent the percentage differences of each parameter obtained to the infected animals (*Bd*^−^) at 0.2 Hz in relation to their respective controls (*Bd*^−^). Significant differences (*P* < 0.05) were observed between species in all the parameters analyzed.

Finally, the values obtained at the stimulation frequency of 0.2 Hz for the infected groups (*Bd*^+^) for each parameter are shown as a percentage of their respective controls (*Bd*^−^) for both species in Fig. 6. Comparatively, chytridiomycosis results in a depressive effect on the CF of *P. albonotatus*, which was 78% lower than that observed to *X. laevis*. This difference between species was even greater (82%) in the cardiac pumping capacity. Regarding the time-dependent parameters, *Bd* infection exerted a much more pronounced effect in *P. albonotatus*, since TPT values were 125% higher than those observed for *X. laevis*, and THR was 57% higher than in *X. laevis*.

## Discussion

Not all amphibian species show adaptive responses to *Bd* infection without having some physiological functions compromised. This can lead to reduced fitness (Pahkala et al., 2003; Bovo et al., 2016), in the case of the cardiac performance itself. *Bd* can be highly virulent and cause mortality in many amphibian species (Nichols et al., 2001; Berger, Speare & Skerratt, 2005; Blaustein et al., 2005). For other species, however, this fungus can infect animals without causing death (Daszak et al., 2004; Weldon et al., 2004; Woodhams et al., 2006; Rosenblum et al., 2013). What in fact determines the tolerance or susceptibility of a species to *Bd* is still unknown (Woodhams et al., 2006). This is due to the lack of studies specifically dedicated to assessing differential physiological effects of this infection in its hosts (Bovo et al., 2016).

Host defenses against pathogens can usually be defined into two types: those that limit pathogen infection intensity by preventing or reducing infection; and those that decrease the fitness consequences of the infection (Venesky et al., 2015). In addition, alterations of physiological parameters may lead to the increased susceptibility to various environmental stressors (Formicki, Zamachowski & Stawarz, 2003).

In this sense, the ability of the heart muscle to maintain its performance, even under adverse physiological conditions, is a characteristic of great adaptive importance to the animals (Driedzic & Gesser, 1994). Hence, in this study, we demonstrate that while *P. albonotatus,* the *Bd-*sensitive amphibian, presented a cardiac impairment; the higher tolerance of *X. laevis* seems to allow this species to develop an adequate cardiac performance in response to chytridiomycosis. Such higher tolerance can imply higher adaptive value for *X. laevis*. Widely sold as pets, this species is considered a potential reservoir and carrier of *Bd* (Weldon et al., 2004; Woodhams et al., 2006; Rollins-Smith et al., 2009). The main factors that seem to promote the tolerance of *X. laevis* to *Bd* infection include immune defenses (Rollins-Smith et al., 2009), and the production of antimicrobial peptides (Woodhams et al., 2006).

In addition, the fact that *X. laevis* has a fully aquatic life cycle may favor tolerance to *Bd* infection because single immune adaptations—that are not shared with terrestrial species—contribute crucially in the adjustments to occasional environmental changes that may occur (Hero, Williams & Magnusson, 2005; Haislip et al., 2011). In contrast, there is a lack of information about whether *P. albonotatus* presents any defensive mechanism to resist or tolerate chytridiomycosis.

The results obtained *in situ* demonstrate that, for *P. albonotatus, Bd* infection results in a negative chronotropic response (i.e., reduction in the heart rate) (Table 1). It is known that adult amphibians’ hearts present a vagal parasympathetic innervation (Burnstock, 1969) that, when stimulated, liberates acetylcholine (ACh). This results in a negative chronotropic response, with reduction of the self-depolarization rate of pacemaker cells (Pelster et al., 1993). Therefore, *Bd* could either stimulate the parasympathetic system, or inhibit the activity of the enzyme acetylcholinesterase (AChE), which is responsible for ACh hydrolysis (Costa et al., 2015). Consequently, ACh accumulates in the synaptic cleft. Among the effects of cholinergic overstimulation previously described in mammals are tearing, sweating, cardiorespiratory alterations, salivation, changes in the central nervous system, bradycardia, as well as muscle necrosis (Nunes, Carvalho & Guilhermino, 2005). Costa et al. (2015) also demonstrated this bradycardia in bullfrog tadpoles exposed to environmental stressors (i.e., exposure to an organophosphate).

Additionally, or alternatively, a decrease in the plasmatic concentration of Na^+^—which is essential to pacemaker cells’ depolarization—can result in the flattening of the self- depolarization curve of this tissue, thereby decreasing the heart rate of *Bd* infected animals. It is worth mentioning that hyponatremia was already demonstrated by Voyles et al. (2009) in adult *Litoria caerulea* exposed to *Bd*.

The infected *P. albonotatus* also presented an atrophy of the heart muscle, as well as lethargy. Again, an inhibition of the AChE activity can cause an overstimulation of the skeletal muscle by acetylcholine, impairing movements. This happens because ACh acts directly on the central nervous system (Nunes, Carvalho & Guilhermino, 2005). However, this lethargy observed in *Bd*-infected *P. albonotatus* can be also a direct consequence of anorexia. Anorexia and lethargy are among the first symptoms presented by *Bd*-sensitive species (Berger et al., 2004; Berger, Speare & Skerratt, 2005; Voyles et al., 2007). It is important to emphasize that the association between lethargy and anorexia causes a metabolic depression and, therefore, results in a decrease on the cardiac demand. This, eventually, may result in heart atrophy (Table 1) (Seakins & Waterlow, 1972; Samarel et al., 1987; Perhonen et al., 2001). Indeed, when subjected to prolonged fasting, animals frequently mobilize amino acids from muscular organs, which can promote cardiac atrophy (Seakins & Waterlow, 1972; Samarel et al., 1987; Castellini & Rea, 1992).

The cardiac atrophy could be one of the causes for the marked decrease in the *in vitro* cardiac performance of *P. albonotatus*, which includes the reduction in the contraction force (Fig. 1A) and in the cardiac pumping capacity (Fig. 3A). Another hypothesis would be that some harmful toxin produced by *Bd* (Brutyn et al., 2012; Fites et al., 2013), once inside the host’s body, could reach the heart. Once in the heart, this toxin could act directly on Ca^2+^ transporting mechanisms through the sarcolemma (slowing Ca^2+^ channels and/or Na^+^/Ca^2+^ exchangers). This impairs Ca^2+^ influx into the cells and causes a negative inotropic effect in infected animals (Driedzic & Gesser, 1985; Gwathmey & Morgan, 1991; Gesser et al., 1997). Indeed, the highest values of TPT and THR (Fig. 4) observed for infected *P. albonotatus* clearly suggest that the prolongation in the cardiac dynamics was associated with changes at the contractile mechanisms (ionic influx and efflux) in myocytes, which were damaged by *Bd* infection. The bradycardia observed *in situ* in the infected animals (Table 1) corroborates these results. As a consequence, *Bd* infection results in a pronounced decrease in the cardiac output in *P. albonotatus*, since both heart frequency and stroke volume (see CPC and CF) were impaired (Withers & Hillman, 2001).

On the other hand, the absence of such symptoms in *X. laevis* reinforces its greater tolerance to chytrid infection. Although for this species there were no changes in the *in situ* heart rate, these animals presented cardiac hypertrophy (Table 1). This represents a homeostatic strategy that could avoid a decrease in the cardiac performance. Such hypertrophy may also be a physiological adjustment in response to the anorexia, in an attempt to rebalance the distribution of nutrients in the organism, through an increase in the systolic volume. Cardiac hypertrophy is frequently observed when increases in cardiac performance are necessary, in response to either pollutants or other factors (Calore, Perez & Herman, 2007). Previous studies with *Lithobates catesbeianus* (also considered a *Bd-*tolerant species) have already demonstrated cardiac hypertrophy in response to *Bd* infection (Salla et al., 2015), which, in turn, could be characterized as a new cardiac biomarker of chytrid tolerance.

The absence of effects on the CF (Fig. 1B) and CPC (Fig. 3B) for *X. laevis* indicates that this species developed some mechanism that reduced the impacts of chytridiomycosis on its cardiac function. Moreover, the lack of changes in TPT and THR of the infected *X. laevis* corroborated the absence of any impairment of the cardiac performance of this species (Fig. 5B). We have previously demonstrated that another *Bd*-tolerant species, *L. catesbeianus*, also develops cardiac adjustments in order to minimize the negative impact of *Bd*-infection, even in tadpoles (Salla et al., 2015).

The correlation between the load of *Bd* acquired by the animals and the contraction force developed by their respective ventricle strips show that there is a species-specific sensitivity pattern in its hosts. (Fig. 2)*.* For *P. albonotatus*, even with infection levels lower than *X. laevis* (Table 2), *Bd* was able to impair its cardiac contractility, which corroborates this species’ greater sensitivity to the chytrid. Indeed, for *P. albonotatus*, the higher the load of infection, the more pronounced the effects of chytridiomycosis on the contraction force, which became drastically reduced. The lack of relationship between *Bd* load and CF for *X. laevis* provides insight into the mechanisms by which this species tolerates the infection. In short, Fig. 6 presents a diagram that clearly illustrates the more pronounced effects of *Bd* infection on the *in vitro* cardiac performance of *P. albonotatus* when compared to *X. laevis*, as previously discussed.

It is important to emphasize that, although an equal spore concentration was used for both species, *Bd* zoospores could be overdispersed across the water column. Thus, as *Physalaemus* specimens were kept in shallow water, and *Xenopus* specimens were kept immersed, the contact with the spores could have been different. This condition may also have contributed to the different levels of infection acquired by each species. Even so, the effects of the infection were evident, especially in *P. albonotatus*, which reinforces the hypothesis that its cardiac function contributes to its greater susceptibility to the disease.

## Conclusions

This is the first study that demonstrates that sensitive amphibian species have their heart function impaired by chytridiomycosis. These effects can result in short-term cardiac failure and subsequent mortality, which affects not only the individual, but also possibly an entire population. Therefore, the sub-lethal effects have an adaptive value for affected species. In contrast, *Bd-*tolerant species such as *X. laevis,* can survive without any impairment of their cardiac system and act as *Bd* reservoirs in the wild*.* Finally, we suggest heart hypertrophy as a possible cardiac biomarker of *Bd* infection in tolerant species. Additonally, the impairment of the contractile and relaxing mechanisms (which lead to cardiac failure) are the direct cause of mortality in *Bd* sensitive hosts.

##  Supplemental Information

10.7717/peerj.5891/supp-1 Supplemental Information 1 Raw data - *Physalaemus albonotatus* The tabs contain the raw data from the *in vitro* and *in situ* experiments of *Physalaemus albonotatus*.

10.7717/peerj.5891/supp-2 Supplemental Information 2 Raw data - *Xenopus laevis* The tabs contain the raw data from the *in vitro* and *in situ* experiments of* Xenopus laevis*.

## Additional Information and Declarations

### Field Study Permissions

The following information was supplied relating to field study approvals (i.e., approving body and any reference numbers): Field experiments were approved by the Brazilian Institute of Environment–IBAMA (collecting permit #18566-1).

### Data Availability

The following information was supplied regarding data availability: The data of this article are available as Supplemental Files and in the repository page of the postgraduate program of Biotechnology and Environmental Monitoring: http://docs.wixstatic.com/ugd/847cf7_e98f3061c39141dc8e99c89599b740a7.pdf.
